# Effects of lowering body temperature via hyperhydration, with and without glycerol ingestion and practical precooling on cycling time trial performance in hot and humid conditions

**DOI:** 10.1186/1550-2783-9-55

**Published:** 2012-12-17

**Authors:** Megan LR Ross, Nikki A Jeacocke, Paul B Laursen, David T Martin, Chris R Abbiss, Louise M Burke

**Affiliations:** 1 Australian Institute of Sport, Belconnen, ACT, Australia; 2 School of Exercise Biomedical and Health Science, Edith Cowan University, Joondalup, Western Australia, Australia; 3 High Performance Sport New Zealand, Auckland, New Zealand; 4 Sports Performance Research Institute New Zealand (SPRINZ), School of Sport and Recreation, AUT University”, Auckland, New Zealand

**Keywords:** Euhydration, Hypothermia, Self-paced endurance performance, Perception of effort, Environmental heat gain, Metabolic heat gain, Heat dissipation

## Abstract

**Background:**

Hypohydration and hyperthermia are factors that may contribute to fatigue and impairment of endurance performance. The purpose of this study was to investigate the effectiveness of combining glycerol hyperhydration and an established precooling technique on cycling time trial performance in hot environmental conditions.

**Methods:**

Twelve well-trained male cyclists performed three 46.4-km laboratory-based cycling trials that included two climbs, under hot and humid environmental conditions (33.3 ± 1.1°C; 50 ± 6% r.h.). Subjects were required to hyperhydrate with 25 g.kg^-1^ body mass (BM) of a 4°C beverage containing 6% carbohydrate (CON) 2.5 h prior to the time trial. On two occasions, subjects were also exposed to an established precooling technique (PC) 60 min prior to the time trial, involving 14 g.kg^-1^ BM ice slurry ingestion and applied iced towels over 30 min. During one PC trial, 1.2 g.kg^-1^ BM glycerol was added to the hyperhydration beverage in a double-blind fashion (PC+G). Statistics used in this study involve the combination of traditional probability statistics and a magnitude-based inference approach.

**Results:**

Hyperhydration resulted in *large* reductions (−0.6 to −0.7°C) in rectal temperature. The addition of glycerol to this solution also lowered urine output (330 ml, 10%). Precooling induced further *small* (−0.3°C) to *moderate* (−0.4°C) reductions in rectal temperature with PC and PC+G treatments, respectively, when compared with CON (0.0°C, P<0.05). Overall, PC+G failed to achieve a clear change in cycling performance over CON, but PC showed a possible 2% (30 s, P=0.02) improvement in performance time on climb 2 compared to CON. This improvement was attributed to subjects’ lower perception of effort reported over the first 10 km of the trial, despite no clear performance change during this time. No differences were detected in any other physiological measurements throughout the time trial.

**Conclusions:**

Despite increasing fluid intake and reducing core temperature, performance and thermoregulatory benefits of a hyperhydration strategy with and without the addition of glycerol, plus practical precooling, were not superior to hyperhydration alone. Further research is warranted to further refine preparation strategies for athletes competing in thermally stressful events to optimize health and maximize performance outcomes.

## Background

During strenuous exercise performed in hot and/or humid conditions, the effects of a high metabolic heat production combined with insufficient heat dissipation lead to the development of hyperthermia [1,2]. These high body temperatures (i.e., >39°C) reduce exercise performance [3,4], as evidenced by the inability to sustain a constant exercise intensity [5,6] or through alterations in self-selected pace [2,7]. Fortunately, there are established strategies that can be applied prior to an event that can lessen the impact of heat gain and facilitate heat loss from the body. For instance, precooling through the application or ingestion/inhalation of cold air, water and ice have been demonstrated to be effective in lowering deep body temperatures and enhancing heat storage capacity (for review, see [8-10]). We have recently established that a combination of external (application of iced towels) and internal (consumption of an ice slurry) cooling is a practical and effective strategy for reducing body temperature and enhancing cycling time trial performance in hot conditions [11,12].

Pre-exercise hyperhydration involves the deliberate intake of large fluid volumes prior to performing an exercise task. This strategy has been proposed to attenuate possible reductions in performance that may occur with dehydration in a hot environment [13]. However, both pre-hydrating [14] and acute cold exposure [15,16] are accompanied by concomitant increases in diuresis, which may limit their usefulness prior to a prolonged event. When compared with water ingestion alone however, fluid retention is increased (~8 ml.kg^-1^ body mass) when osmotically active agents such as sodium or glycerol are consumed with the fluid [13]. Furthermore, the addition of glucose to a solution containing glycerol may further enhance fluid absorption and be of further benefit from a metabolic perspective [17]. A recent meta-analysis concluded that the use of glycerol hyperhydration in hot conditions provides a small (3% power output, Effect Size=0.35) but worthwhile enhancement to prolonged exercise performance above hyperhydration with water [13]. However, some studies involving glycerol hyperhydration have failed to show performance benefits [18-22] and furthermore, it appears that the beneficial effects may not be simply explained in terms of an attenuated body fluid deficit. Rather, improved exercise performance may be the result of a reduction in body temperature with glycerol hyperhydration [18,23,24].

In light of the unknown but potentially interrelated effects of precooling and pre-exercise hyperhydration, with and without glycerol, on endurance performance, the present study aimed to investigate the effectiveness of combining glycerol hyperhydration and an established precooling technique on cycling time trial performance in hot environmental conditions. In addition, a sub-purpose was to examine this objective using high levels of construct validity, by using as many real-life competition circumstances as possible, such as a high pre-exercise environmental heat load and a simulated performance trial with hills and appropriate levels of convective cooling.

## Methods

### Subjects

Twelve competitive well-trained male cyclists (mean ± SD; age 31.0 ± 8.0 y, body mass (BM) 75.2 ± 9.2 kg, maximal aerobic power (MAP) 444 ± 33 W, peak oxygen consumption ($\dot{V}$ O_2peak_) 68.7 ± 8.8 ml.kg^-1^.min^-1^) were recruited from the local cycling community to participate in this study. Prior to commencement of the study, ethical clearance was obtained from the appropriate human research ethics committees. Subjects were informed of the nature and risks of the study before providing written informed consent. Prior to the study, subjects completed a medical questionnaire and had no prior history of heat intolerance, current injury or illness.

### Study overview

On separate days following heat acclimation and an incremental exercise test to exhaustion, participants performed a total of three hilly 46.4-km experimental cycling time trials (described below) in hot environmental conditions (33.3 ± 1.1°C; 50 ± 6% r.h.). Three trials were conducted in a randomized counterbalanced order. Prior to the commencement of all performance trials (t=−180 min), subjects were required to ingest 25 g.kg^-1^ BM of a cold (4°C) beverage containing 6% carbohydrate (CHO; Gatorade, Pepsico, Australia, NSW, Australia). Additionally, on two occasions, subjects were also exposed to an established combined external and internal precooling technique, whereby iced towels were applied to the subject’s skin while ingesting additional fluid in the form of an ice slurry (slushie) made from sports drink (PC). The precooling method used in this study, as previously described [11], commenced 60 min prior to the start of the trial (t=−60 min) and was applied for a period of 30 min. During one of the precooling trials, the recommended dose [25] of 1.2 g.kg^-1^ BM glycerol (PC+G) was added to the large fluid bolus in a double blind fashion. PC and PC+G trials were compared to a control trial, which consisted of the large beverage ingestion without glycerol and received no precooling (CON). Experimental trials were separated by 3–7 d with a consistent recovery time between trials for each subject.

### Heat acclimation

Prior to the first experimental trial, subjects visited the laboratory on at least nine occasions to heat acclimate and familiarize with the cycle ergometer (Velotron, Racermate Inc., Seattle, WA, USA) and the experimental exercise protocol (simulated Beijing Olympic time trial course as previously described [11]). Heat acclimation was completed over a three-week period and consisted of prolonged (>60 min) sub-maximal self-paced cycling, which was performed on at least nine occasions. All acclimation sessions were conducted in a heat chamber under climatic conditions (32-35°C, 50% r.h.) similar to the experimental trials (described below). In addition to the heat acclimation trials, all subjects completed at least one familiarization trial of the experimental cycling protocol in the heat chamber.

### Incremental cycle test

Prior to the first experimental trial subject’s maximal aerobic power (MAP) and peak oxygen consumption ($\dot{V}$ O_2peak_) were characterized by performing a progressive maximal exercise test on a cycle ergometer (Lode Excalibur Sport, Groningen, The Netherlands) as previously described [11].

### Experimental time trials

Subjects followed a standardized pre-packaged diet and training schedule for 24 h prior to each experimental trial. The standardized diet was supplied in the form of pre-packaged meals and snacks, providing 9 g.kg^-1^ BM CHO; 1.5 g.kg^-1^ BM protein; 1.5 g.kg^-1^ BM fat, with a total energy goal of 230 kJ.kg^-1^ BM. Subjects refrained from any intake of caffeine and alcohol over this period. Individualized menus were prepared accounting for food preferences using FoodWorks Professional Edition (Version 6.0, Xyris Software, Brisbane, Australia), as described previously [26]. Subjects were provided with all foods and drinks in portion controlled packages for the first 20 h of the standardized period and were given verbal and written instructions on how to follow the diet. Subjects were allowed to undertake light exercise on the day prior to each trial and were asked to repeat this for subsequent trials. Compliance to the diet and exercise protocol was determined from a checklist kept by each subject and presented on arrival to the laboratory prior to each trial. Subjects’ ‘first-waking’ urine sample was also analyzed for the determination of specific gravity to ensure the cyclist attended the laboratory for each trial in a similar hydration state.

For each experimental trial subjects were required to cycle a 46.4-km time trial on a Velotron cycle ergometer, (Velotron 3D Software, RacerMate Inc., Seattle, WA, USA) which was fitted with a calibrated [27] SRM cycling power meter (scientific version, 8 strain gauge, Schoberer Rad Meβtechnik; Jülich, Germany), which was set to sample at 1 s intervals. The measurement error for cycling time trials during laboratory protocols such as this has been established as 1.7%, as described previously [11]. The course profile for this time trial was a simulation of the 2008 Beijing Olympic Games time trial course, as described previously [11]. All experimental trials were carried out in the afternoon, to mimic the schedule of the 2008 Olympic Games cycling time trial. On arrival to the laboratory, three hours prior each trial (t=−180 min), subjects voided their bladder (not for collection) and inserted a single use thermal probe (Mon-a-therm General Purpose Temperature Probe, Mallinckrodt Medical Inc., St Louis, MO, USA) 12 cm beyond the anal sphincter for determination of rectal temperature (T_re_). Changes in rectal temperature at the end of the precooling phase (t=−30 min) and at the end of the warm-up phase (t=0 min) were used to reflect the effectiveness of the precooling treatment and the potential differential for heat storage at the commencement of the time trial. Reduction in rectal temperature as a result of precooling were categorized as either small (<0.3°C), moderate (0.3-0.6°C), large (0.6-0.8°C) or very large (>0.8°C) based on our previous work [11].

On arrival at the laboratory, subjects were immediately given a large cold beverage (given as two boluses of 12.5 g.kg^-1^ BM at t =−180 and −165 min) to consume within 30 min. At t=−150 min and every 30 min leading up to the commencement of the time trial, and immediately afterwards, subjects were required to void their bladder. Urine was weighed and analyzed for specific gravity. At this time, subjects consumed the last of their standardized diet as a “pre-race meal” which provided 2 g.kg^-1^ BM CHO.

Rating of thermal comfort, T_re_ and HR (Polar S810i HR monitor; Polar Electro OY, Kempele, Finland) were recorded before entering the heat chamber, and every 5 min during 60 min of passive rest in the heat chamber (heat stabilization; t=−120 to −60 min). The environmental conditions inside the chamber were measured and corrected every 5 min throughout the duration of the trial. On two occasions (PC and PC+G trials), following the completion of the stabilization phase, subjects consumed 1,024 ± 122 g slushie containing 6% CHO, which was equivalent to 13.6 g.kg^-1^ BM, providing a CHO intake of 61 g (0.8 g.kg^-1^ BM). The slushie was given in two ~7 g.kg^-1^ BM boluses and subjects were given 15 min to consume each bolus while wearing iced towels, as previously described [11]. During the control trial subjects received no cooling intervention (CON). During this time subjects were also asked to provide ratings of stomach fullness.

Following stabilization and precooling, subjects completed a standardized 20-min warm-up on the Velotron ergometer. The warm-up consisted of two bouts of 3 min at 25% MAP, 5 min at 60% MAP and 2 min at 80% MAP, which is a protocol used by some elite time trial cyclists prior to competition. The final 10 min before the start of the time trial allowed subjects to complete their own preparations. During this time subjects were provided with standard pre-race instructions and the zero offset of the SRM crank was set according to manufacturer’s instructions.

Feedback provided to the subject was limited to distance covered (km), cycling gear-ratio (12-27/42-54), road gradient (%) and instantaneous velocity (km.h^-1^). Subjects were provided with 314 ± 207 g fluid containing 6% carbohydrate (Gatorade, Pepsico Australia, Chatswood, Australia), which provided a further CHO intake of 19 g (0.25 g.kg^-1^ BM) at the “top of each climb” (12.5 and 37.5 km), which simulated the ideal time to consume fluid on the Beijing time trial course based on the experience of professional cyclists during training and racing on the actual course. On the first trial, subjects were given a total of 325 ml at each of these points and were permitted to drink *ad libitum* for the next kilometer on the first trial. The volume that was consumed was measured and repeated for subsequent trials. Drinks were removed from ice storage at the commencement of the time trial and left in the heat chamber to simulate drink temperatures that would be experienced in race conditions. To further replicate competition, the cyclist was positioned in front of a large industrial fan (750 mm, 240 V, 50 Hz, 380 W, model Number: N11736, TQ Professional), which was adjusted to simulate uphill or downhill wind speeds. Specifically, the fan was fixed on low speed to simulate 12 km.h^-1^ wind speed for 0–12.5; 23.2 - 35.7 km and switched to high speed to simulate 32 km.h^-1^ wind speed for 12.5 -23.2 and 35.7 - 46.4 km.

Split times, velocity and power output data were collected for each trial, with the periods of interest being time to top of first climb (12.5 km), end of first lap (23.2 km), time to top of second climb (35.7 km) and finish (46.4 km). Throughout the trials, HR and T_re_ were recorded every 2 min, while self-reports of perception of effort [28], thermal sensation [29], and gastrointestinal comfort (5-point Likert scale), were recorded at approximately 5-km intervals. On the completion of each time trial, subjects were asked a series of questions related to their effort, motivation, sensation and comfort, as reported previously [11].

### Statistical analysis

Pre-trial body mass, percentage dehydration, and post-trial subjective ratings were compared between trials (i.e., CON, PC, PC+G) using a one-way analysis of variance (ANOVA). A two-way (trial × time) repeated measures ANOVA was used to examine differences in dependant variables (i.e., rectal temperature, heart rate, urine specific gravity and volume, thermal comfort, stomach fullness and RPE) between trial means at each time point. If a significant main effect was observed, pairwise comparisons were conducted using Newman-Keuls *post hoc* analysis. These statistical tests were conducted using Statistica for Microsoft Windows (Version 10; StatSoft, Tulsa, OK) and the data are presented as means and standard deviations (SD). For these analyses, significance was accepted at P<0.05.

The performance data from the three trials were analysed using the magnitude-based inference approach recommended for studies in sports medicine and exercise sciences [30]. A spreadsheet (Microsoft Excel), designed to examine post-only crossover trials, was used to determine the clinical significance of each treatment (available at newstats.org/xPostOnlyCrossover.xls), as based on guidelines outlined by Hopkins [31]. Performance data are represented by time trial time and power output during the various segments of the course, and are presented as means ± SD. The magnitude of the percentage change in time was interpreted by using values of 0.3, 0.9, 1.6, 2.5 and 4.0 of the within-athlete variation (coefficient of variation) as thresholds for small, moderate, large, very large and extremely large differences in the change in performance time between the trials [30]. These threshold values were also multiplied by an established factor of −2.5 for cycling [32], in order to interpret magnitudes for changes in mean power output. The typical variation (coefficient of variation) for road cycling time trials has been previously established as 1.3% by Paton and Hopkins [33], with the smallest worthwhile change in performance time established at 0.4% [34], which is equivalent to 1.0% in power output. These data are presented with inference about the true value of a precooling treatment effect on simulated cycling time trial performance. In circumstances where the chance (%) of the true value of the statistic being >25% likely to be beneficial (i.e., faster performance time, greater power output), a practical interpretation of risk (benefit:harm) is given. An odds ratio (OR) of >66 was used to establish that the benefit to performance time gained by using one strategy outweighed any potential harm (in performance time) that could result.

## Results

### Performance

Performance time (h:min:s) and power output (W) for the entire time trial, for each of two laps and for each of four segments (climb 1 and 2, and descent 1 and 2) of each time trial are presented in Table 1. Overall performance time and average power output were not significantly different between any of the three performance trials (P>0.05). However, there was a possibility of performance benefits on selected parts of the course. On Lap 2 of the PC condition, there was a 1.2% reduction in performance time (30 s; P=0.07) and a 1.4% increase in power output (3 W, P=0.34) compared with CON. This improvement was brought about by the 1.8% faster performance time (30 s; P=0.02) and greater power output (6 W, P=0.07) that was achieved predominantly on the climbing section (Climb 2). Moreover, the likelihood of a detrimental performance outcome was sufficiently outweighed by the chance of benefit (OR>66).

**Table 1:** Summary of cycling time trial performance data: performance time and power output

**Course Profile**		**Treatment**	**Performance time**			**Power output**			**Qualitative inference**
**Phase**	**Distance**	**Intervention**	**mean ± SD**	**Mean Δ; ± 90% CL**	**P**	**mean ± SD**	**Mean Δ; ± 90% CL**	**P**	**(% Chance of positive / trivial / negative outcome compared to CON)**
	**(km)**		**(h:min:sec.0)**	**(%)**		**(W)**	**(%)**		
Total	0 – 46.4	CON	1:18:47 ± 5:09	-	-	276 ± 37	-	-	-
		PC	1:18:28 ± 4:40	−0.4; ± 0.9	0.49	277 ± 34	0.5; ± 2.0	0.66	Unclear (4/96/0)
		PC+G	1:18:47 ± 5:10	0.0; ± 1.5	0.99	278 ± 40	0.5; ± 3.7	0.79	Unclear (7/87/6)
		(PC V PC+G)	-	−0.4; ± 1.2	0.60	-	0; ±3.2	0.99	Unclear (8/91/1)
Lap 1	0 – 23.2	CON	38:55 ± 2:23	-	-	279 ± 36	-	-	-
		PC	39:06 ± 2:23	0.5; ± 1.3	0.55	277 ± 36	−0.6; ± 2.2	0.63	Unclear (21/84/14)
		PC+G	39:17 ± 2:34	0.9; ± 1.5	0.31	276 ± 41	−1.3; ± 3.3	0.51	Unclear (1/66/32)
		(PC V PC+G)	-	−0.4; ± 1.3	0.54	-	0.7; ± 3.3	0.72	Unclear (13/86/2)
Lap 2	23.2 – 46.4	CON	39:52 ± 2:50	-	-	273 ± 39	-	-	-
		PC	39:22 ± 2:28	−1.2; ± 1.1	0.07	276 ± 33	1.4; ± 2.6	0.34	Possible improvement (31/69/0); OR>66
		PC+G	39:29 ± 2:45	−0.9; ± 2.0	0.41	278 ± 43	2.4; ± 5.2	0.41	Unclear (30/68/2); OR<66
		(PC V PC+G)	-	−0.3; ± 1.7	0.78	-	−0.6; ± 4.5	0.82	Unclear (11/85/4)
Climb 1	0 – 12.5	CON	25:46.6 ± 1:58.1	-	-	289 ± 31	-	-	-
		PC	25:55.6 ± 1:59.0	0.6; ± 1.7	0.54	291 ± 37	0.4; ± 2.5	0.77	Unclear (2/84/14)
		PC+G	26:03.8 ± 2:09.2	1.1; ± 2.1	0.39	291 ± 42	0; ± 3.8	0.99	Unclear (2/66/32)
		(PC V PC+G)	-	−0.5; ± 1.6	0.61	-	0.4; ± 3.1	0.81	Unclear (11/87/2)
Climb 2	23.2 – 35.7	CON	26:56.7 ± 2:22.0	-	-	274 ± 39	-	-	-
		PC	26:26.2 ± 2:05.5	−1.8; ± 1.2	0.02	280 ± 33	2.4; ± 2.1	0.07	Possible improvement (49/51/0); OR>66
		PC+G	26:36.9 ± 2:21.0	−1.2; ± 2.4	0.37	280 ± 43	2.8; ± 4.7	0.29	Unclear (33/65/2); OR<66
		(PC V PC+G)	-	−0.6; ± 2.2	0.63	-	−0.1; ± 4.6	0.97	Unclear (16/80/3)
Descent 1	12.5 – 23.2	CON	13:08.7 ± 35.2	-	-	254 ± 38	-	-	-
		PC	13:10.3 ± 32.3	0.2; ± 0.8	0.65	251 ± 35	−1.0; ± 3.1	0.56	Unclear (1/91/7)
		PC+G	13:13.3 ± 36.2	0.6; ± 0.9	0.25	248 ± 41	−2.4; ± 4.9	0.38	Likely trivial (0/77/23)
		(PC V PC+G)	-	−0.4; ± 0.9	0.49	-	1.4; ± 4.2	0.56	Unclear (14/85/1)
Descent 2	37.5 – 46.4	CON	12:54.9 ± 37.3	-	-	270 ± 42	-	-	-
		PC	12:55.7 ± 32.3	0.1; ± 0.8	0.78	267 ± 35	−0.6; ± 4.1	0.80	Unclear (1/95/4)
		PC+G	12:52.5 ± 35.3	−0.3; ± 1.1	0.63	273 ± 44	1.8; ± 6.4	0.61	Unclear (13/84/3)
		(PC V PC+G)	-	0.4; ± 0.7	0.29	-	−1.7; ± 4.8	0.53	Likely trivial (0/92/8)

Note: CL = confidence limits; OR = odds ratio; P = probability; Outcomes were assessed by using the following criteria: trivial <0.4%, small 0.4 – 1.1%, moderate 1.2-2.0%, large 2.1-3.2%, very large 3.3 – 5.1%, and extremely large >5.2% change in performance time.

**Figure 1:**
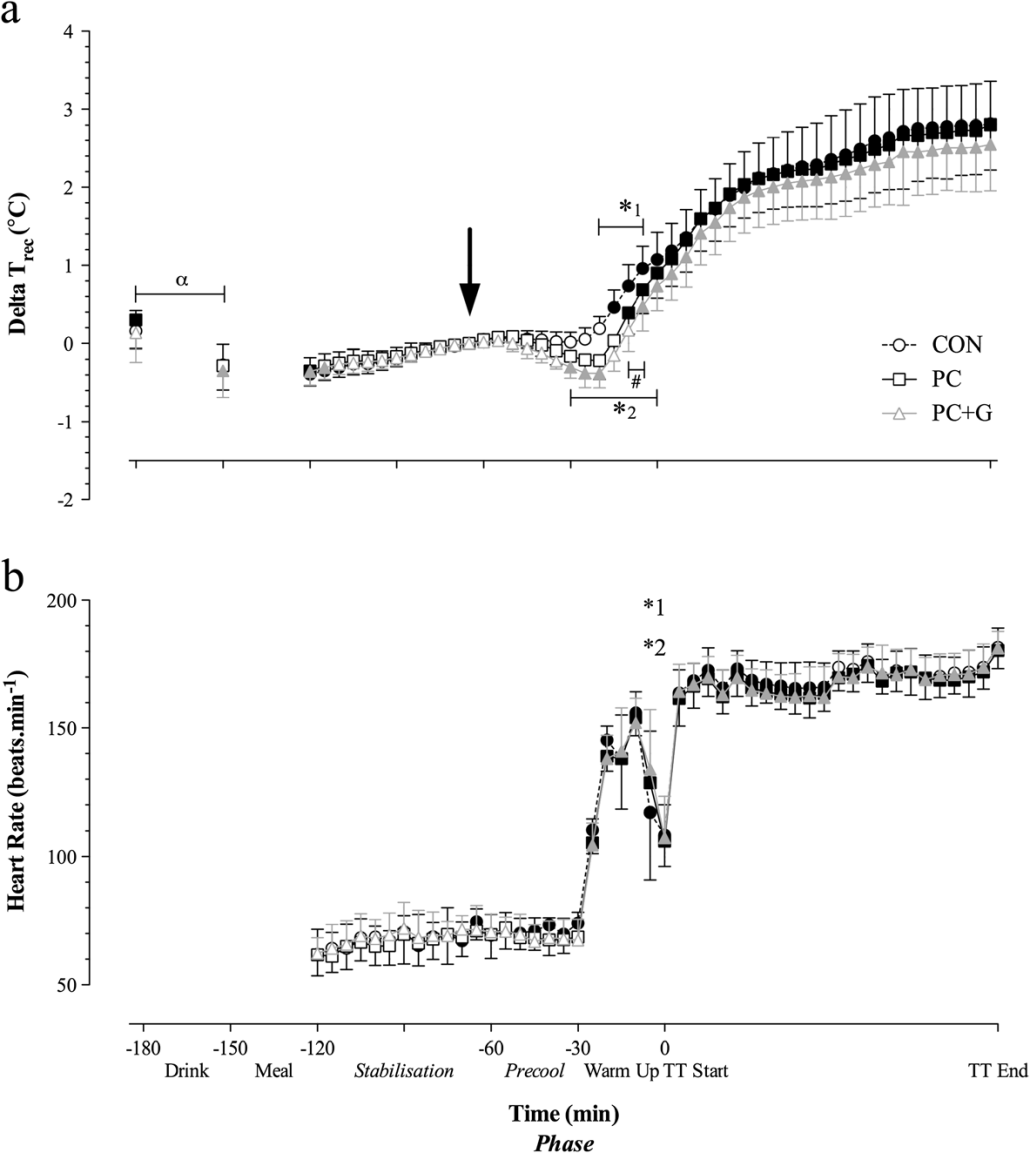
**Relative change in rectal temperature (a) and heart rate (b) throughout the experimental trial. **Significant time effects from t=−65 min before TT (arrow) are denoted by *dark symbols*. Significant time effect from t=−180 min to t=−150 min following drink ingestion with and without glycerol ingestion denoted by alpha (α). Significant effects of precooling treatment (1; PC and 2; PC+G) compared with CON are denoted by a star symbol (*_1_,*_2_, respectively). Significant interaction between PC and PC+G treatments are denoted by a hash (#) symbol.

Rectal temperature towards the end of the stabilization phase (t=−65 min before the TT) was considered to be the baseline value for each trial. At this time point, there were no differences in rectal temperature between trials (P>0.05, Figure 1a). Relative change in rectal temperature at the end of the warm-up and just prior to the time trial was significantly lower in the PC+G compared with the CON trial (P<0.05). Relative change in rectal temperature continued to rise during the time trial in all trials, such that there was no difference in relative change in rectal temperature between treatments during this phase (CON, 1.33 ± 0.27°C.h^-1^; PC, 1.45 ± 0.32°C.h^-1^; PC+G, 1.39 ± 0.26°C.h^-1^; P>0.05). Figure 1b shows the changes in heart rate during each trial.

**Figure 2:**
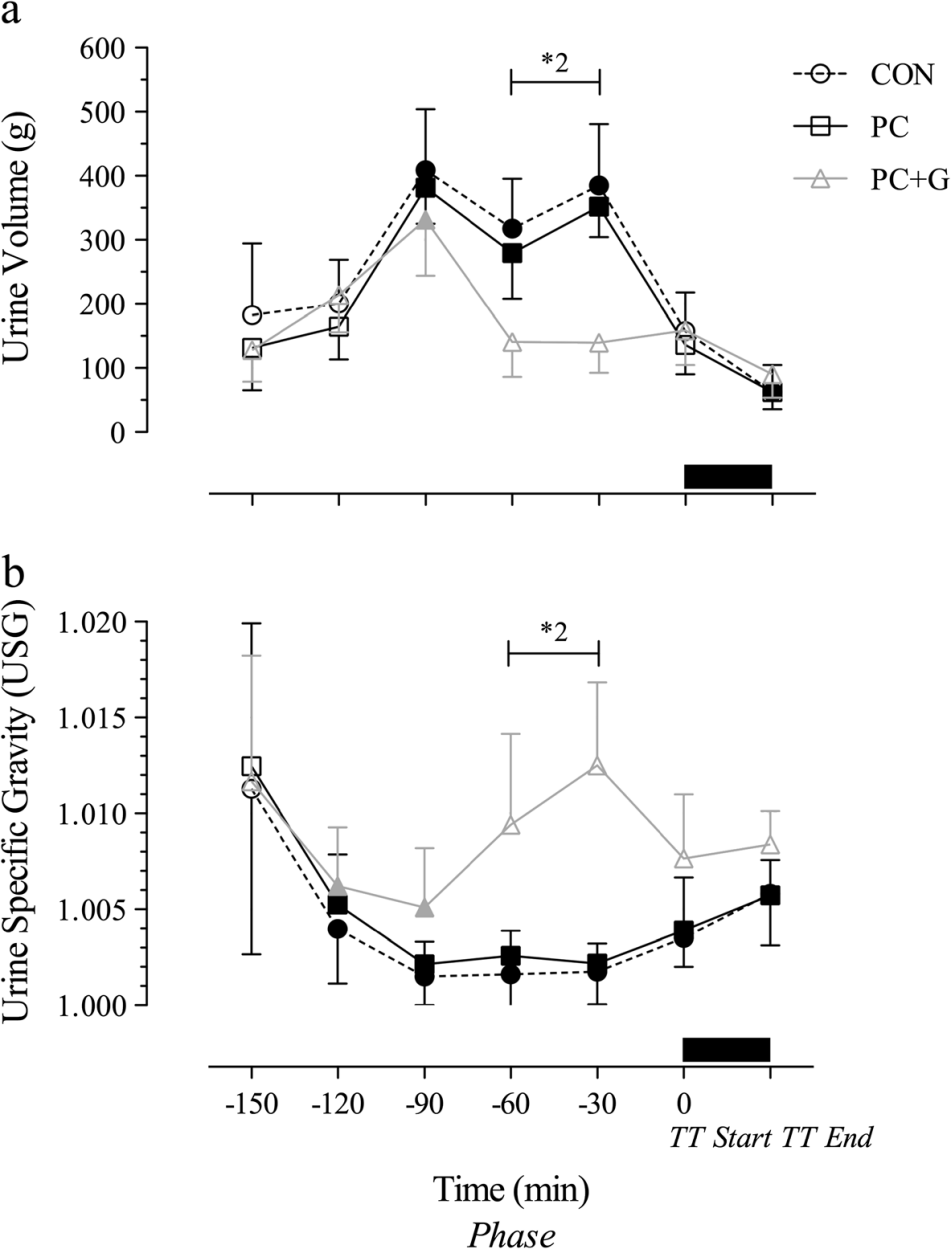
**Volume of urine output (a) and urine specific gravity (b) throughout the experimental trial. **Significant time effects from t=−150 min before TT are denoted by *dark symbols*. Significant treatment effect of PC+G compared with CON denoted with star symbol (*_2_). Time trial denoted by black bar.

Collection of ‘first-waking’ urine samples on the morning of each trial, mean changes in body mass, fluid consumed and urine volume produced during the trials are presented in Table 2. The time course of urine production represented in Figure 2a and the corresponding specific gravity of these samples is represented in Figure 2b. Due to the inclusion of slushie ingestion being part of the precooling intervention, the amount of sports drink ingested by subjects inside the heat chamber (t=−120 min to end of the time trial or ~3.5 h) was greater in PC (1,335 ± 211 ml) and PC+G (1,356 ± 206 ml) trials, compared with the CON (299 ± 214 ml, P<0.001) trial, which provided a further ~80 g of carbohydrate.

**Table 2:** Fluid balance

	**CON**	**PC**	**PC + G**
	**Mean ± SD**	**Mean ± SD**	**Mean ± SD**
‘First waking’ Urine Specific Gravity	1.015 ± 0.005	1.015 ± 0.005	1.016 ± 0.004
Δ BM^A ^(kg)	−2.56 ± 0.60	−2.50 ± 0.61	−2.52 ± 0.60
Δ BM^A ^(%)	−3.19 ± 0.83	−3.13 ± 0.90	−3.14 ± 0.85
Sweat rate ^A ^(L.h^-1^)	−1.94 ± 0.48	−1.91 ± 0.48	−1.92 ± 0.47
Total fluid consumed ^B ^(L)	2.18 ± 0.74	3.22 ± 1.24*	3.24 ± 1.25*
Total urine volume ^C ^(L)	1.71 ± 0.34	1.51 ± 0.30	1.20 ± 0.36 *^#^

Note: ^A ^represents n=11; pre to post time trial, B represents fluids consumed from −180 min prior to the time trial until the end of the time trial, ^C ^represents urine volume collected from −150 min prior to the time trial until immediately after the time trial, * represents substantial difference to CON (P<0.05), ^# ^represents substantial difference between PC and PC+G treatments (P=0.03).

**Figure 3:**
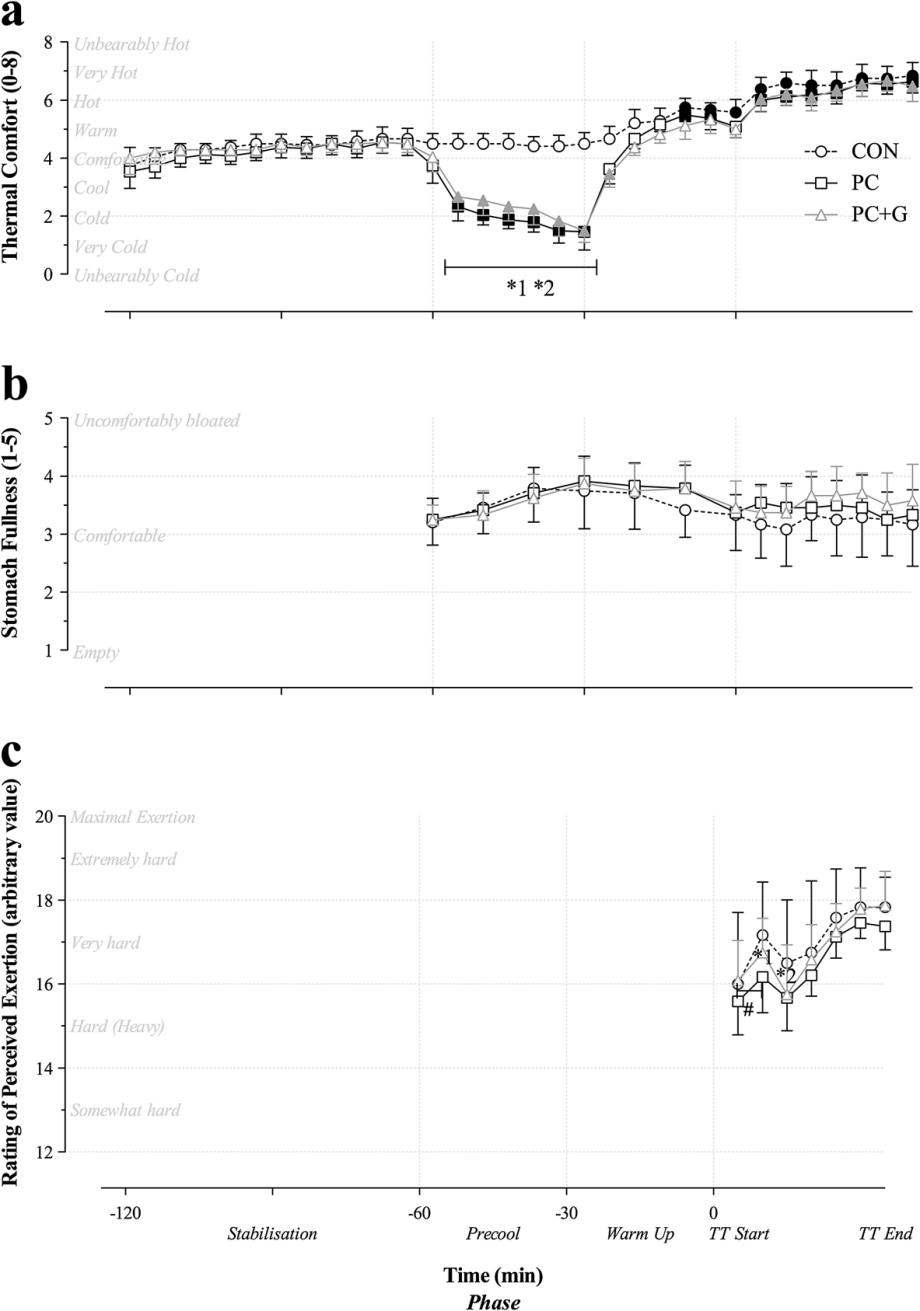
**Subjective ratings of comfort. **Thermal comfort (**a**), stomach fullness (**b**). and rating of perceived exertion (**c**). Significant time effects from t=−65 min before TT are denoted by *dark symbols*. Significant effects of precooling treatment (1; PC and 2; PC+G) compared with CON are denoted by a star symbol (*_1_,*_2_, respectively).

There was no significant change in the rating of thermal comfort after subjects had entered the heat chamber to stabilize to the hot and humid conditions for 60 min (t=−120 to −60 min pre TT, Figure 3a). However, once precooling commenced (t=−60 min before the time trial), the rating of thermal comfort was significantly reduced, such that subjects reported feeling cooler when treated with PC and PC+G (t=−55 to −25 min before time trial, P<0.05). There was no significant change in ratings of perceived stomach fullness (Figure 3b) across the three trials, however, there were significant interactions (P<0.05, Figure 3c) detected in RPE throughout the first 17 km of the time trial (Climb 1 and the first 4.5 km of descent 1).

Subjective information provided by each subject at the completion of each trial are presented in Table 3. These data suggest that subjects’ perceived level of effort, sensations, motivation and comfort experienced, were similar across all trials.

**Table 3:** Subjective information on completion of time trials

**Theme**		**CON**			**PC**			**PC + G**		
		**(mean ± SD)**			**(mean ± SD)**			**(mean ± SDcpa**		
Effort given	(%)	94	±	10	95	±	6	98	±	4
Sensation	(Arbitrary value)	4.0	±	0.9	3.8	±	1.1	3.8	±	0.8
Motivation	(Arbitrary value)	4.6	±	1.4	4.9	±	1.2	5.2	±	0.7
Comfort	(Arbitrary value)	2.4	±	1.2	2.5	±	0.9	2.9	±	0.7

Note: All comparisons P>0.05.

## Discussion

The purpose of the current study was to investigate the effectiveness of combining glycerol hyperhydration and a practical precooling strategy on performance during a cycling time trial that simulated a real-life event in hot and humid environmental conditions. The main findings of this study were that: i) a hyperhydration strategy, with or without the addition of glycerol, in addition to an established precooling technique, failed to achieve a clear enhancement of cycling time trial performance in hot humid conditions, ii) the ingestion of a large volume of chilled (4°C) fluid prior to the time trial (CON) induced a clear and sustained *large* reduction in body temperature, and iii) when precooling, involving the application of iced towels and the ingestion of a slushie, was performed after consumption of a hyperhydration solution without, but not with glycerol, a further “small” reduction in deep body temperature, reduced perceived exertion and improved performance on the second half of the time trial (i.e., climb 2) occurred.

Our original hypotheses were that our precooling strategy would result in lower body temperatures compared with the control condition and the prior ingestion of a hyperhydration strategy would be further enhanced with the addition of glycerol. While glycerol hyperhydration resulted in an increased fluid balance of ~330 ml (10%) and the precooling technique caused a further *small* to *moderate* reduction in deep body temperature, together these alterations did not lead to a clear improvement in overall performance. In fact, on further inspection of performance data, a *possible* (49% chance) performance benefit (2%) was observed on climb 2 following hyperhydration, without glycerol, plus precooling (PC intervention) over the control trial. This improved performance was associated with subjects reporting a lower perception of effort over the first 10 km of the time trial (2.5 km short of the top of the climb), despite similar pacing strategies and physiological perturbations (i.e., rectal temperature, heart rate, thermal comfort and stomach fullness) across all trials. As such, it appears that benefits associated with hyperhydration plus precooling offered some advantage in attenuating the perception of effort during the initial portion of the trial, allowing for improved performance in the later stages of the trial when thermal load was greatest. These results may be partially explained by the pre-trial brief, in which subjects were instructed “if feeling *good*, to save the *big* effort for the second lap”.

Despite lower core body temperature and improved thermal comfort as a result of precooling and hyperhydration with the co-ingestion of glycerol, performance was not significantly different to the control trial over any section of the course. Moreover, although subjects received the same precooling intervention, the magnitude of cooling was greater in the PC+G trial compared with the PC trial (a *moderate* versus *small* reduction in rectal temperature, respectively). We are unable to provide a clear explanation into the potential mechanism of this enhanced effect. However, the differences in performance among trials in the present study, despite differing core body temperatures are commensurate with those from our previous (unpublished) observations, whereby a greater reduction in rectal temperature did not lead to greater performance effects. These results thus provide further data to refute the existence of a direct relationship between magnitude of cooling and the functional outcome [8,35]. In fact, we may have induced a magnitude of cooling that surpassed a threshold temperature, in which performance may be impaired during self-paced endurance exercise, however this currently remains speculative.

While results of the present study may indicate that the precooling and hyperhydration interventions used are ineffective in enhancing real life sporting performance, an unexpected finding from this study was that the ingestion of the pre-event fluid in the control trial, also induced a clear and sustained *large* reduction in body temperature. A chilled beverage was selected as the control condition for hyperhydrating subjects to mask the flavor characteristics of the glycerol in the sports drink in PC+G trial, to standardize total fluid intake, and to simulate the conditions of a real-life event. Indeed, when performing in hot and humid conditions, participants are usually exposed to the environmental conditions for more than 2 hr prior to the event and in most circumstances would preferentially ingest a cool beverage. It is possible that the *large* reduction in rectal temperature observed in the control trial may have provided a benefit to performance and thus reduced the likelihood of observing clear physiological or performance effects. Indeed, this protocol and magnitude of cooling observed is similar to studies that have shown improvements in endurance capacity following cold fluid ingestion precooling [36-38]. These studies used ~20.5 to 22.5 ml.kg^-1^ fluid served at 4°C in the 90 min before [36] and/or during [37,38] an exercise task performed in hot and humid conditions. Interestingly, we observed a sustained cooling effect with mean baseline rectal temperature (t=−65 pre time trial) remaining below pre-hydration levels, despite subjects being exposed to the hot and humid conditions for ~60 min following consumption. Although we cannot determine whether the reduction in core body temperature improved performance in the present study, we have previously shown that the same precooling strategy resulted in a 3% increase in average cycling power output of similar calibre cyclists over the same course [11], when compared to a control trial without any fluid intake. Collectively these results indicate that hyperhydration with or without glycerol, plus precooling through the application of iced towels and the ingestion of a slushie, may provide minimal performance benefit, over the ingestion of a large cool beverage.

Although the focus of precooling was the optimization of thermoregulation, we acknowledge the composition of the slushie, in the current study, provided additional fluid and carbohydrate; nutritional components that may also enhance performance. However, as we have previously discussed [11,12], it is unlikely that performance of our cycling protocol would be influenced by providing euhydrated subjects with further fluid or having greater carbohydrate availability associated with this strategy, at least within the limits of detection of our protocol and under the control conditions of nutritional preparation (i.e., following a carbohydrate rich mean, well hydrated). Furthermore, this study design was representative of real-life circumstances, whereby cyclists simply added the precooling strategy to a hyperhydration strategy.

In summary, the current study does not support the hypothesis that hyperhydration, with or without the addition of glycerol, plus an established precooling strategy is superior to hyperhydration, in reducing thermoregulatory strain and improving exercise performance. Despite increasing fluid intake and reducing core body temperature, hyperhydration plus precooling failed to improve performance when compared with the consumption of a large cool beverage prior to the trial. These results indicate that a combined precooling technique (i.e., ice towel application and slushie ingestion) results in minimal performance benefit over and above the typical real-life pre-race preparations (i.e., consumption of a cold fluid). Further research is warranted in order to examine the influence of fluid temperature and volume on the success of glycerol hyperhydration and precooling strategies, presumably because the control condition, chosen to standardize total fluid intake, also involved a substantial precooling effect. Specifically, further studies could be undertaken to compare glycerol hyperhydration using a tepid beverage to distinguish the effects of this strategy on fluid status from its thermoregulatory impact and allow separation of the different elements that may underpin a performance change.

## Competing interests

The authors declare that they have no competing interests.

## Authors’ contributions

All authors have made substantive intellectual contributions towards conducting the study and preparing the manuscript for publication. All authors read and approved the final manuscript. Specifically, MR was involved in concept and design of the study, gaining ethical clearance, subject recruitment, acquisition of the data, preparing tables and figures for publication, interpretation of the data and all aspects of writing the manuscript; NJ was responsible for dietary standardisation and dispensing nutritional interventions, contributed to preparation of methodology, assisted with data analysis and review of manuscript; DTM was responsible for concept and design of the study, analysis and interpretation of data, and revision of the manuscript; PL was responsible for concept and design of the study, interpretation of data, and revision of the manuscript; CA was involved in concept and design of the study, acquisition and interpretation of the data, drafting of the manuscript; LB was responsible for securing funding for the study, concept and design of the study, overseeing ethical submission, data interpretation and drafting of the manuscript.
